# Performance of Ultrasound-guided Peripheral Nerve Blocks by Medical Students After One-day Training Session

**DOI:** 10.7759/cureus.3911

**Published:** 2019-01-18

**Authors:** Elaine H Situ-LaCasse, Richard Amini, Victoria Bain, Josie Acuña, Kara Samsel, Christina Weaver, Josephine Valenzuela, Landon Pratt, Asad E Patanwala, Srikar Adhikari

**Affiliations:** 1 Emergency Medicine, University of Arizona, Tucson, USA; 2 Emergency Medicine, Banner University Medical Center, Tucson, USA; 3 Pharmacology, University of Arizona, Tucson, USA

**Keywords:** point-of-care ultrasound, peripheral nerve blocks, medical student education, ultrasound-guided procedures, medical simulation, medical education, pain management, pain management, opioid addiction

## Abstract

Introduction

Ultrasound-guided peripheral nerve blocks (USGPNB) are performed by various specialists and are excellent, non-addicting pain control techniques. Alternative pain management approaches are needed to combat opiate abuse. Medical students should be aware of alternative pain management therapies before they begin clinical practice.

Objective

Our objective was to determine if medical students can identify peripheral nerves under ultrasound and perform a USGPNB after a one-day hands-on training session.

Methods

This was a cross-sectional study at an academic medical center. The study participants were third-year medical students with minimal prior ultrasound experience. Students were given an introductory lecture highlighting the opiate epidemic and benefits of USGPNB prior to the workshop. The one-day hands-on educational workshop consisted of learning basic sonographic anatomy, indications for USGPNB, and practicing needle guidance under ultrasound guidance. After the educational workshop, students’ procedural competency was assessed by ultrasound-trained emergency medicine clinicians.

Results

A total of 94 participants were included in this study. The average pre-test score was 68.4% (95% confidence interval [CI]; 65.4% to 71.4%). After the one-day educational workshop, the post-test score was 92.8% (95% CI; 90.8% to 94.8%). The average hands-on evaluation score was 84.4% (95% CI; 81.6% to 87.3%). All students agreed that this educational session is a good start to learning about USGPNB, and they felt comfortable identifying the peripheral nerves using ultrasound. On a confidence scale of one (low) through 10 (high), 83% (95% CI; 75.9% to 90.15%) rated their confidence as ≥6. All except one student either agreed that this educational session helped them understand how USGPNB could be integrated into acute pain management. The majority (84% [95% CI; 77% to 91%]) agreed that the session will change how they manage patients’ acute pain in their future medical practice.

Conclusion

Medical students can learn the sonographic anatomy of peripheral nerves and techniques of USGPNB after a one-day educational session.

## Introduction

Ultrasound-guided peripheral nerve blocks (USGPNB) have been performed for decades by pain specialists, anesthesiologists, and emergency medicine physicians [1] and are easy to learn and perform [2]. Ultrasound guidance for peripheral nerve blocks improves the success rates [3]. In the peri-operative time period, USGPNB requires less analgesia supplementation or conversion to general anesthesia [4]. When ultrasound is used, the time to procedure completion was less than that taken for landmark-based blocks (5 minutes vs. 9.8 minutes) with decreased complications [4-5].

As more medical schools are integrating ultrasound into their curricula, educators need to integrate more timely, advanced applications of ultrasound into medical student education [6]. Ultrasound-guided procedures are becoming more commonplace, and the hand-eye coordination and muscle memory development are important to initiate in medical school. In addition to learning this technique, it is also critical to introduce other methods of pain management to medical students to shift the emphasis away from opioids as the first line of defense against pain, especially in our current opioid epidemic [7]. Our objectives were to 1) determine if medical students can identify peripheral nerves under ultrasound and 2) perform a USGPNB after a one-day hands-on training session.

## Materials and methods

Study design and setting

This was a single-site cross-sectional study conducted at an academic medical center. The study falls under the University of Arizona College of Medicine’s umbrella institutional review board (IRB) as approved education research according to the pre-determined requirements of the College of Medicine’s original IRB approval. The study was offered to all third-year medical students, and ultimately, we had 94 third-year medical students with minimal prior ultrasound experience who completed the entire study. They were three months into their third year of medical school, which is their first year of clinical medicine rotations. Participation in this study was voluntary, and data were collected in September 2017. Students were taught and assessed by the Emergency Ultrasound fellowship-trained clinicians, the Emergency Ultrasound fellows, or the Emergency Medicine residents.

Educational curriculum

**Figure 1:**
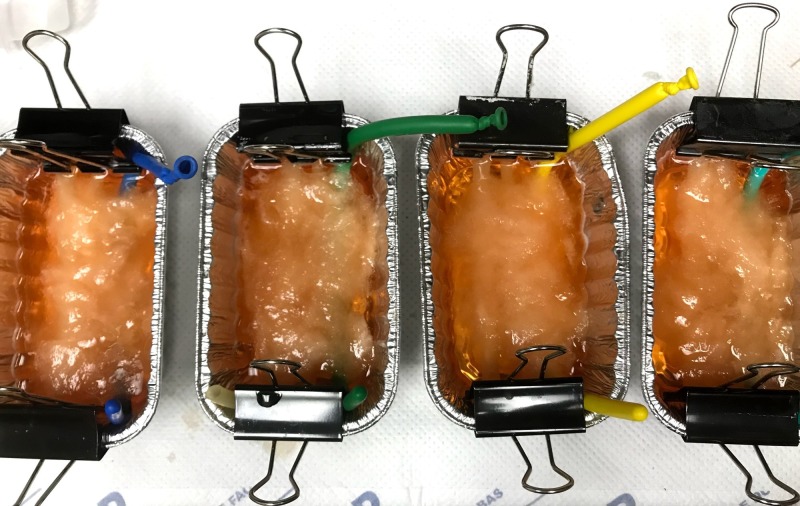
Nerve block phantoms

**Figure 2:**
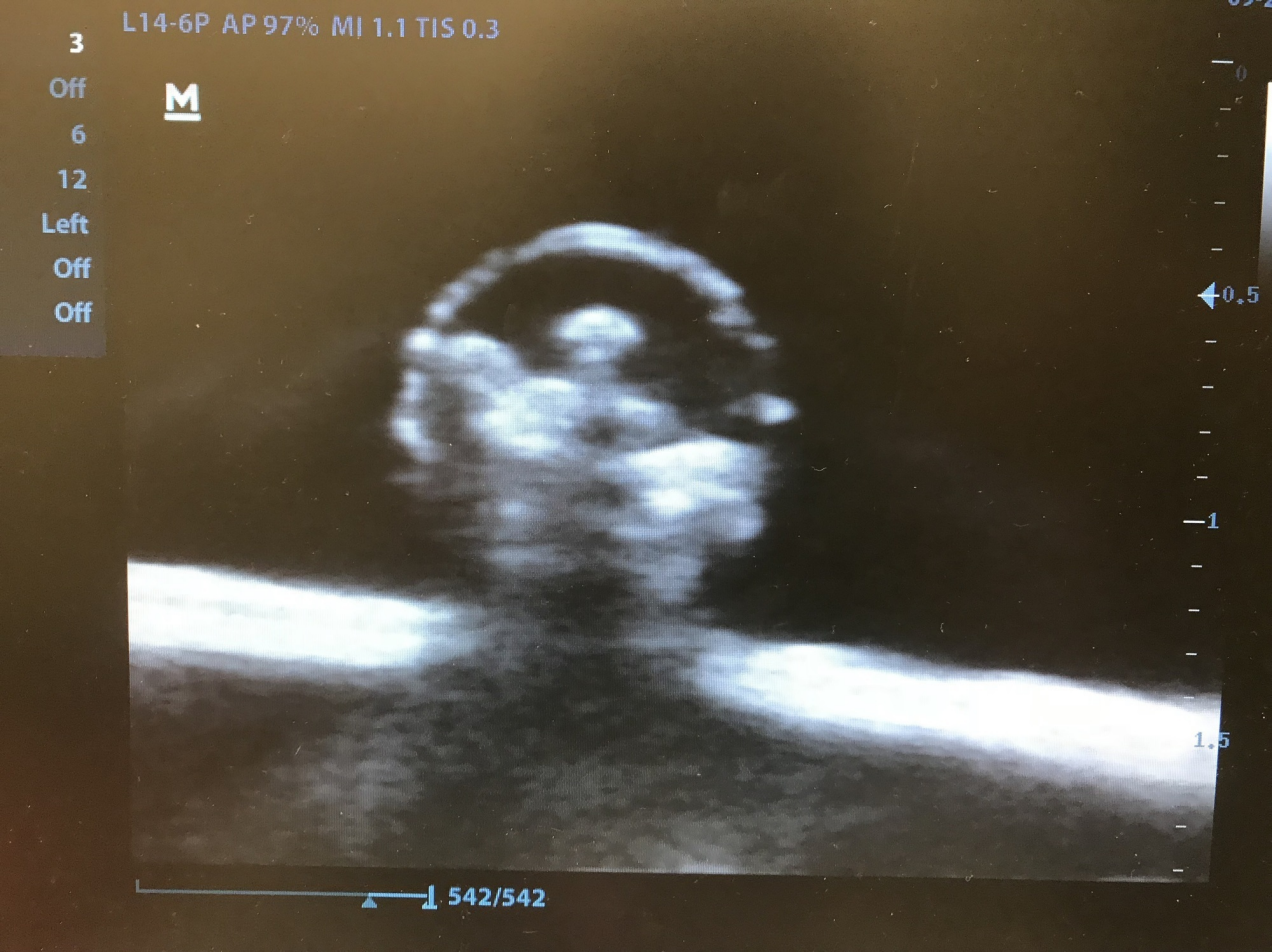
Simulated nerve under ultrasound

Prior to the workshop, students were given a one-hour didactic lecture highlighting the opioid epidemic and introducing the benefits of USGPNB for pain management. Students were asked to complete a pre-test prior to the one-hour didactic. No other demographic information or prior experience data was collected. The next day, during the one-day educational workshop, students were taught basic sonographic musculoskeletal and nerve anatomy of upper and lower extremities on live models, as seen in Table 1. The educational portion was 2.5 hours. Specifically, the students were taught how to identify the median, ulnar, radial, femoral, and sciatic nerves with ultrasound. Students also received instructions regarding the indications for USGPNB and examples of USGPNB, and then they were provided approximately 45 minutes during the educational portion to practice needle guidance under ultrasound using a nerve block phantom. Identical nerve block phantoms were created using latex tubing of two differing thicknesses, simulating vein and artery. The simulated nerve was created by inserting uncooked angel hair pasta into the lumen of long balloons and filling them with a small volume of water. These three structures were then set in ballistic gel and covered with cotton to obscure the structures. Similar models have been developed by our ultrasound section in the past. Please refer to Figures 1-2. Students then rotated from a hands-on teaching station to a hands-on assessment station.

Assessment

The assessment consisted of a questionnaire post-test identical to the pre-test, hands-on assessment, and exit survey. The pre-test questionnaire was administered prior to the one-hour didactic and participation in the session. The post-test and exit surveys were completed immediately after the hands-on assessment. The identical pre-test/post-test included medical knowledge-based questions regarding basic ultrasound physics, nerve anatomy and distribution, and ultrasound-guided nerve block protocols. The exit survey queried opinions regarding the effectiveness of the educational session, pain management, and confidence in USGPNB. Descriptions of the stations and objectives have been provided in Table 1.

**Table 1:** Skills station descriptions and objectives

Session Descriptions	Objectives
Educational session – 2.5 hours	Probes and Knobology:
	-Explain the differences between the various probes
	-Know the use of a linear probe for nerve blocks
	-Learn the Optimize button function
	-When/how color Doppler is used
	Upper extremity nerves:
	-Identify the median, radial, and ulnar nerves
	-Find nerves’ neighboring arteries
	-Demonstrate anisotropy in nerves
	-Identify tendons and nerves
	-Describe differences between tendons and nerves
	-Know nerve sensory distributions
	Lower extremity nerves:
	-Identify femoral nerve and sciatic nerve
	-Identify branches of the sciatic nerves
	-Identify veins vs arteries with compression
	-Identify veins vs arteries with color Doppler
	-Identify neurovascular bundle in femoral area
	-Identify the neurovascular bundle in the popliteal area
	-Know nerve sensory distributions
	Nerve block phantom (~45 minutes for practice):
	-Identify the supplies needed for the procedure
	-Visualize needle under ultrasound in the long axis
Assessment session – 1.2 hours	Hands-on evaluation (live model and phantom)
	Completion of post-test and exit survey

Statistical analysis

Descriptive statistics were used to analyze the data. Continuous data were presented as averages with standard deviations and 95% confidence intervals, and dichotomous data and nominal data were presented as a percentage of frequency of occurrences.

## Results

A total of 94 participants were included in the data analysis for the assessments and hands-on session evaluation. The average pre-test score was 68.4% (95% confidence interval [CI]; 65.4% to 71.4%). After the one-day educational workshop, the post-test score was 92.8% (95% CI; 90.8% to 94.8%). The average hands-on evaluation score was 84.4% (95% CI; 81.6% to 87.3%). Scores are summarized in Table 2. A total of 106 anonymous exit surveys were completed. It appeared that more students completed the survey, but did not complete the assessments. Since the surveys were anonymous, these could not be matched to the assessments. All students either strongly agreed or somewhat agreed that this educational session was “a good start to learning about USGPNB,” and 97.1% (95% CI; 93.9% to 100.3%) “felt comfortable identifying the median, ulnar, radial, femoral, and sciatic nerves using ultrasound.” On a confidence scale of one (low) through 10 (high), 5.7% (95% CI; 1.3% to 10.1%) rated their confidence as three or less, and 70.6% (95% CI; 61.9% to 79.3%) rated it seven or above. All except one student agreed that this educational session helped them understand how USGPNBs could be integrated into acute pain management. The majority (84% [95% CI; 77% to 91%]) agreed that the session will change how they manage patients’ acute pain in their future medical practice. 

**Table 2:** Test scores CI: confidence interval

	Average scores % (95% CI)	Score range % (lowest score to highest score)
Pre-test	68.4 (65.4 to 71.4)	18.2 to 90.9
Post-test	92.8 (90.8 to 94.8)	45.5 to 100
Hands-on evaluation	84.4 (81.6 to 87.3)	35.7 to 100

## Discussion

Ultrasound education has gained significant traction in medical school training as it has been shown to improve student understanding of anatomy, clinical skills development, and clinical decision-making [8]. Early exposure to ultrasound education has been shown to improve ultrasound procedural skills by the end of training [9-11]. Other procedures, such as paracenteses and thoracenteses, have also seen improvement in patient safety and complications through the use of ultrasound guidance [12]. PNBs are also an example of a procedure that has benefited from ultrasound guidance. The use of ultrasound during PNBs can reduce pain during procedures, decrease complications, increase patient satisfaction, and reduce the need for rescue analgesia [2]. 

We report a one-day hands-on session curriculum used to educate medical students on the importance of alternative pain management options, sonographic anatomy, and USGPNB skill development. Previous studies have demonstrated the integration of ultrasound into the medical school curriculum and the ability of students to learn ultrasound-guided needle guidance [13-15]. However, this is the largest group of medical students to learn USGPNB. Given the current state of prescription drug use and abuse [7], it is imperative that alternative pain control measures be evaluated and taught. To our knowledge, no studies have looked at the medical students’ ability to learn and perform USGPNB. Our results demonstrate that the students were able to successfully identify nerves using ultrasound.

Amini et al. demonstrated a significant improvement between pre-test and post-test of MS3 students in ultrasound-guided procedures after a short didactic session [15]. Similarly, we found moderate improvement in our study between the pre-test and post-test. Similar to previous literature, our results showed increased procedural confidence after this educational session [15]. Our one-day session was well-received, with students stating that the session was a good start to learning USGPNB. By introducing advanced concepts early, it becomes possible for medical students to integrate them into practice later. Ultrasound education and learning ultrasound-guided procedures serve as a foundation for all future physicians even if they are not performing nerve blocks in their practice. Our study has shown that it is feasible for the students to acquire basic ultrasound skills in a one-day workshop and then apply them with proficiency in their future career. 

With our educational ultrasound session, the medical students not only learned how to perform USGPNBs but were also introduced to the importance of knowing this technique as the medical field is trying to treat and prevent opioid addiction. Majority of the medical students responded in their exit survey that this educational session helped them understand how USGPNB could be integrated in treating acute pain. Most of the students also stated that this educational session changed how they will manage acute pain in their future clinical practice. 

Limitations

Our study has several limitations. The educational curriculum was not pilot tested or validated prior to implementation. Our study included instructors with varied levels of ultrasound experience (clinicians, fellows, and residents) which could have contributed to the variation in our study outcomes. 

Students' hands-on assessment did not have a pre-test comparator. We also did not evaluate for skills retention. There is degradation of well-documented skills after just six weeks [16]. This study was designed only to assess hands-on skills right after the educational training session. 

Also, a number of students had completed the pre-test, post-test, and exit survey. Because these activities were anonymous, the students could not be tracked individually. 

The nerve block phantom was an adequate replacement for a live model for procedural practice; but nonetheless, the feel was far from the experience of performing a nerve block on a live patient.

## Conclusions

Medical students should be aware of alternative pain management therapies before they begin clinical practice. Our objective was to determine if medical students can identify peripheral nerves under ultrasound and perform a USGPNB after a one-day hands-on training session. The results showed that their medical knowledge of USGPNBs increased along with their confidence in performing the procedure and in understanding the utility of USGPNB as an adjunct or replacement to opiate pain medication. We conclude that medical students can learn the sonographic anatomy of peripheral nerves and techniques of USGPNB after a one-day educational session. Our study shows that it is feasible for medical schools to incorporate USGPNBs into a curriculum that discusses pain management.

## Appendices

USGPNB Education Pre-Test and Identical Post-Test

1.   Anisotropy is:

a.    When a sound pulse bounces back and forth between two strong parallel reflective structures

b.    A setting on the machine that measures velocity of blood flow through a vessel

c.    The artifact seen when gas is trapped in subcutaneous tissues

d.    A tissue property that is responsible for a structure’s changes in echogenicity depending on the angle of the ultrasound beams

e.    A preset on the ultrasound machine to auto-adjust the brightness of the ultrasound image

2.    If a nerve block were to be performed on the nerve shown below [median nerve under ultrasound], what symptoms will the patient experience?

a.    Anesthesia of the palmar surface of thumb, index, and middle fingers

b.    Anesthesia of palmar surface of the fifth finger

c.    Anesthesia of the dorsal surface of the forearm

d.    Anesthesia of the majority of the dorsal surface of the hand

e.    None of the above

3.    Which of the following are possible complications of nerve blocks?

a.    Nerve damage

b.    Intravascular injection

c.    Hematoma

d.    Lidocaine toxicity

e.    All of the above

4.    True or False: Linear array probes emit higher frequency ultrasound waves, so it is best used to visualize superficial structures such as peripheral nerves.

5.    In-plane needle guidance:

a.    Allows one to visualize the entire needle, including its tip

b.    Allows one to see the needle in the short-axis view

c.    Allows one to see the needle in the long-axis view

d.    Is unnecessary in ultrasound-guided procedures

e.    Both a and c

6.    True or False: When ultrasound is used for nerve block guidance, less anesthetic is necessary compared to the anatomical landmarks-only technique.

7.    The honeycomb appearance of a nerve under ultrasound is secondary to:

a.    The arteries and veins

b.    Individual neurons

c.    Neuronal fat

d.    Perineurium surrounding fascicles

e.    Schwann cells

8.    When one is attempting to find the femoral nerve under ultrasound, where is the nerve typically located in relation to the femoral artery?

a.    Medial to the artery

b.    Lateral to the artery

c.    Anterior to the artery

d.    Posterior to the artery

e.    Cannot visualize the artery and nerve at the same time

9.    Patient presents with a fracture of their fifth metacarpal head after punching a wall. Which nerve do you block? 

a.    Radial nerve

b.    Ulnar nerve

c.    Sural nerve

d.    Median nerve

e.    Sciatic nerve

10.  Your elderly patient presents to the Emergency Department with a femoral neck fracture, which nerve should you block to give the patient good pain relief?

a.     Femoral nerve

b.    Tibial nerve

c.    Common peroneal nerve

d.    Sciatic nerve

e.    Obturator nerve

11.  A football player of the university college team comes in with an Achilles tendon rupture. He wants some pain relief but does not want to take narcotic pain medications. Which nerve block should you perform?

a.    Femoral nerve

b.    Tibial nerve

c.    Common peroneal nerve

d.    Sciatic nerve

e.    Obturator nerve

USGPNB Education Exit Survey

1.  To what extent do you agree or disagree with this statement: “This SonoCamp is a good start to learning about ultrasound-guided nerve blocks.” (Circle one.)

a.    Strongly agree

b.    Somewhat agree

c.    Neither agree nor disagree

d.    Somewhat disagree

e.    Strongly disagree

2.   To what extent do you agree or disagree with this statement: “I feel comfortable identifying the median, ulnar, radial, femoral, and sciatic nerves using ultrasound.” (Circle one.)

a.    Strongly agree

b.    Somewhat agree

c.    Neither agree nor disagree

d.    Somewhat disagree

e.    Strongly disagree

3.  Please rate your confidence level in performing an ultrasound-guided nerve block. (Circle one number.)

Low                                                                                                     High

1          2          3          4          5          6          7          8            9          10

4.  To what extent do you agree or disagree with this statement: “SonoCamp helped me understand how ultrasound-guided nerve blocks could be effectively integrated into the management of patients with acute pain.” (Circle one.)

a.  Strongly agree

b.  Somewhat agree

c.  Neither agree nor disagree

d.  Somewhat disagree

e.  Strongly disagree

5.  To what extent do you agree or disagree with this statement: “The ultrasound learning sessions over the past 2 days will change how I manage patients’ acute pain in my future medical practice.” (Circle one.)

a.  Strongly agree

b.  Somewhat agree

c.  Neither agree nor disagree

d.  Somewhat disagree

e.  Strongly disagree
